# Nanosheet-Stabilized Emulsions: Near-Minimum Loading and Surface Energy Design of Conductive Networks

**DOI:** 10.1021/acsnano.1c06519

**Published:** 2022-02-02

**Authors:** Sean P. Ogilvie, Matthew J. Large, Marcus A. O’Mara, Anne C. Sehnal, Aline Amorim Graf, Peter J. Lynch, Adam J. Cass, Jonathan P. Salvage, Marco Alfonso, Philippe Poulin, Alice A. K. King, Alan B. Dalton

**Affiliations:** † University of Sussex, Brighton BN1 9RH, United Kingdom; ‡ University of Brighton, Brighton BN2 4GJ, United Kingdom; § Centre de Recherche Paul Pascal - CNRS, University of Bordeaux, 33600 Pessac, France

**Keywords:** emulsions, graphene, molybdenum disulfide, liquid phase exfoliation, surface energy

## Abstract

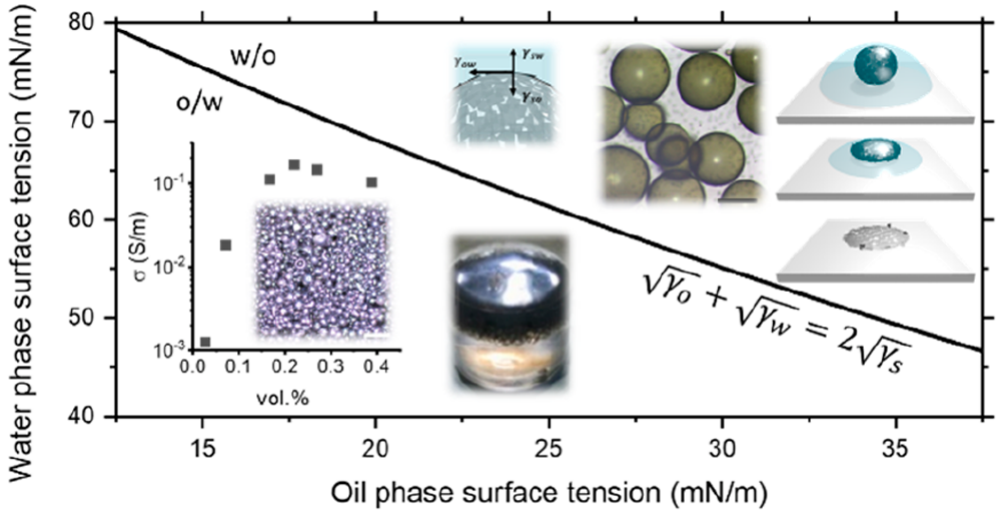

Here, we develop a framework for assembly, understanding, and application of functional emulsions stabilized by few-layer pristine two-dimensional (2D) nanosheets. Liquid-exfoliated graphene and MoS_2_ are demonstrated to stabilize emulsions at ultralow nanosheet volume fractions, approaching the minimum loading achievable with 2D materials. These nanosheet-stabilized emulsions allow controlled droplet deposition free from the coffee ring effect to facilitate single-droplet devices from minute quantities of material or assembly into large-area films with high network conductivity. To broaden the range of compositions and subsequent applications, an understanding of emulsion stability and orientation in terms of surface energy of the three phases is developed. Importantly, this model facilitates determination of the surface energies of the nanosheets themselves and identifies strategies based on surface tension and pH to allow design of emulsion structures. Finally, this approach is used to prepare conductive silicone emulsion composites with a record-low loading level and excellent electromechanical sensitivity. The versatility of these nanosheet-stabilized emulsions illustrates their potential for low-loading composites, thin-film formation and surface energy determination, and the design of functional structures for a range of segregated network applications.

Two-dimensional (2D) nanosheets, such as graphene and molybdenum disulfide (MoS_2_), are particularly promising materials for the assembly of nanostructured networks with a range of electronic, electrochemical, thermal, and mechanical properties and potential for versatile solution processing.^1^ The objective of such network formation is typically to preserve nanosheet functionality and exfoliation while producing macroscopic structures with low nanosheet volume fraction. In principle, this can be achieved by assembling nanosheets into few-layer conductive films within controlled structures where the high aspect ratio of exfoliated nanosheets enables macroscopic connectivity and thereby conductivity at low loading levels. While macroscopic nanosheet networks have been limited to random polymer composites^2^ or templated segregated networks with reduced percolation threshold,^3−5^ it would clearly be beneficial to assemble such networks in liquid to assemble few-layer films with controlled structure and composition to broaden the range of accessible applications.

Interfacial assembly in liquids is a versatile approach to assemble ultrathin films by ensuring their energetic confinement between two immiscible phases.^6−8^ Such interfaces can be exploited for macroscopic assembly of nanosheets as Pickering emulsions, where nanosheets stabilize droplets of one liquid phase in another.^9^ Clearly, for 2D nanosheets, this presents a route to preserving few-layer films on droplets that can be assembled into macroscopic networks, with the nanosheets acting as both emulsion stabilizer and functional filler. Their few-layer nature and correspondingly high specific surface area could allow stabilization of microscale droplets with nanoscale film thickness, potentially enabling functional networks close to a minimum loading achievable with 2D nanosheets.

While Pickering emulsification has been studied for clays,^10−12^ graphene oxide (GO),^13−15^ reduced GO,^16^ and graphitic multilayers,^17−21^ resulting in either nonconductive structures or in conductive networks with thick interfacial films that require high loadings, emulsions stabilized by pristine few-layer nanosheets have not yet been realized. Here, we develop a framework for understanding, design, and application of emulsions stabilized by pristine few-layer nanosheets to demonstrate their potential for ultralow loading conductive networks, controlled thin-film deposition, and fundamental interface science.

## Results and Discussion

### Exfoliation and Emulsification

**Figure 1:**
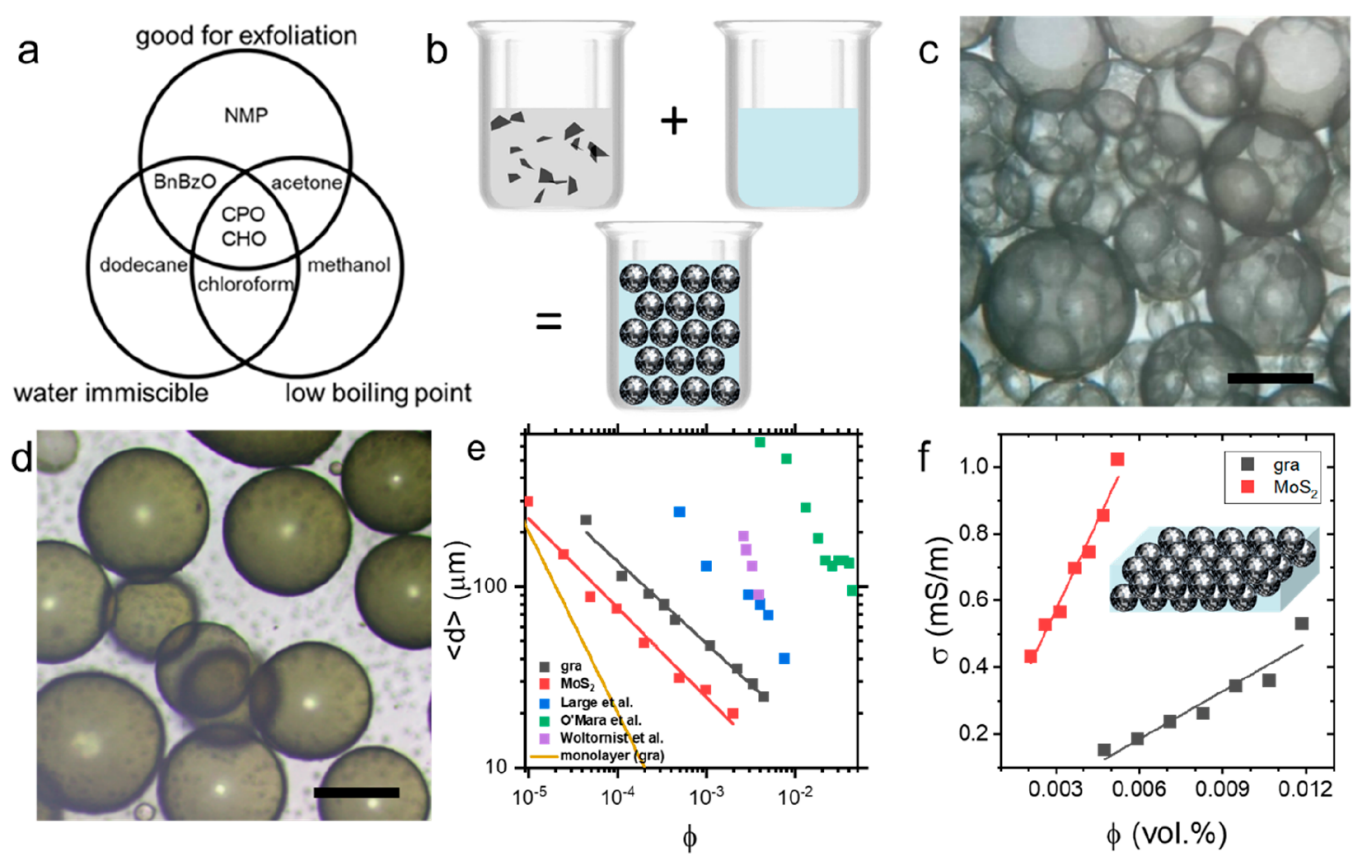
(a) Venn diagram illustrating solvent selection considerations for nanosheet-stabilized emulsions. (b) Schematic diagram of emulsification process where pristine few-layer nanosheets in a dispersion are homogenized with an immiscible liquid to form an emulsion and illustration of nanosheets on the surface of a droplet. (c) and (d) Optical micrographs of water-in-cycloketone droplets stabilized by graphene and MoS_2_, respectively; scale bar 100 μm. (e) Droplet diameter as a function of the nanosheet volume fraction for graphene and MoS_2_ emulsions showing a comparison to previous work and the minimum loading level defined by monolayer graphene. (f) Electrical conductivity of liquid emulsions as a function of nanosheet volume fraction for graphene/H_2_O/CHO and MoS_2_/H_2_O/CPO with an inset schematic of the droplet network.

In order to realize emulsions stabilized by few-layer nanosheets, it is first necessary to perform exfoliation in appropriate solvents. Since emulsions are composed of a high surface tension “water” phase, most often water in which 2D nanosheets cannot be dispersed without surfactant^22^ or additional treatment,^23^ and a low surface tension “oil” phase, which can be any water-immiscible organic, the most obvious route to formation of these emulsions is exfoliation into this oil phase followed by emulsification with water. As such, the selection criteria for appropriate solvents, which allow exfoliation, are water immiscible, and ideally low boiling point for subsequent removal, can be illustrated as in Figure 1a. The requirement for exfoliating solvents that are well-matched in surface energy and Hansen parameters to the nanosheets^1,24,25^ precludes water-immiscible organics such as chloroform, ethyl acetate, and common monomers such as methyl methacrylate. Moreover, the requirement for water immiscibility also precludes common exfoliating solvents such as *N*-methyl-2-pyrrolidone, dimethylformamide, acetone, and most alcohols. Using this solvent selection approach, illustrated in Figure 1a, cyclopentanone (CPO) and cyclohexanone (CHO) were identified as water-immiscible solvents for direct exfoliation and emulsification, which also have relatively low boiling points to facilitate subsequent evaporation. We find higher concentrations (∼0.1 g/L) and stability for graphene with CHO and MoS_2_ with CPO and use these as standard exfoliating solvents for these materials. These dispersions are characterized by statistical Raman mapping using established metrics^26,27^ to confirm their few-layer nature and determine nanosheet size (see Supporting Information Figure S1).

These cycloketone dispersions facilitate the formation of nanosheet-stabilized emulsions by addition of deionized water, as illustrated in Figure 1b, followed by simply shaking by hand. This yields emulsions, as shown in Figure 1c,d, with droplet diameters between 10 and 500 μm and semitransparency indicating stabilization by thin interfacial films. The formation of stable droplets indicates that the requirement for partial wetting of the nanosheet stabilizer by both liquid phases has been satisfied. In addition, this highlights that despite these cycloketones being good solvents for exfoliation, facilitating exfoliation to few-layer nanosheets at reasonable concentrations, the emulsion structure gives a lower energy configuration of the system. This indicates that these pristine nanosheets are neither hydrophilic nor as hydrophobic as the cycloketone oil phase, making them ideal for emulsion formation. Furthermore, the sedimentation of these droplets evidence the formation of water-in-oil (w/o) emulsions as expected and previously demonstrated for pristine nanosheets, indicating some degree of preferential wetting by the oil phase.

This sedimentation also illustrates the potential of these emulsion droplets as building blocks of segregated networks where high nanosheet coverage ensures connectivity and conductivity of thin interfacial films on microscale droplets to realize low-loading networks. As such, the relationship between droplet size and nanosheet volume fraction both indicates interfacial film thickness and informs the functional properties of the resultant networks. To characterize this, the water-in-cycloketone emulsions were formed with a fixed ratio of liquids but varying nanosheet volume fraction and average droplet diameter measured by statistical optical microscopy. Figure 1e shows the average droplet diameter as a function of volume fraction with values between 10 and 500 μm for nanosheet loadings between 1 vol % and 0.001 vol %. Importantly, this highlights that such an approach enables the formation of nanosheet-stabilized emulsions at ultralow volume fractions. This can be interpreted using a simple geometric relation equating the nanosheet specific surface area and droplet surface area to estimate the area-averaged interfacial film thickness as number of monolayers ⟨*N*⟩:
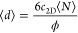
1where ϕ is the volume fraction of the nanosheets relative to the droplet phase and *c*_2D_ is the interlayer spacing in the bulk material (full derivation in Supporting Information). Estimation of these interfacial film thicknesses yields area-averaged values as low as 5 for graphene and 0.3 for MoS_2_ (see Supporting Information Figure S3), indicating that these emulsions are approaching the monolayer limit and corresponding minimum loading level attainable with 2D nanosheets. Interfacial film thicknesses increase sub-linearly with increasing nanosheet volume fraction, likely indicating overcoating of interfaces during emulsification, resulting in increased loading without a corresponding decrease in droplet size. In addition, the differences between the size-loading relationship in graphene and MoS_2_ are likely the result of differences in solvent viscosity, nanosheet size, and edge interactions rather than inherent limitations on interfacial film thickness. While this model provides only an estimate of interfacial film thickness, these droplet sizes are comparable to previous studies of pristine graphitic emulsions, but achieved at significantly lower loading levels and approaching the monolayer limit, as shown in Figure 1e. This highlights the influence of the exfoliation approach in realizing few-layer interfacial films on microscale droplets for macroscopic networks.

Importantly, these water-in-cycloketone emulsions also facilitate system-scale conductivity enabled by conduction pathways across interfacial nanosheet films and tunneling through interdroplet cycloketone layers. These conductivities are shown in Figure 1f increasing with nanosheet volume fraction, suggesting an increased interfacial film thickness or parallelization of the conductive network dominates over the increased density of interdroplet junctions. Interestingly, the MoS_2_ emulsion networks show a higher conductivity than the graphene emulsion networks, suggesting the emulsion conductivity is influenced by interdroplet junctions and network structure (MoS_2_ droplets are smaller at same volume fraction, see Supporting Information Figure S3). These liquid-based conductive networks represent, to the best of our knowledge, the lowest-loading macroscopic pristine nanosheet networks ever reported. This highlights nanosheet-stabilized emulsions as promising low-loading functional structures with potential for the composition and structure to be tuned for specific applications.

### Controlled Droplet Deposition

**Figure 2:**
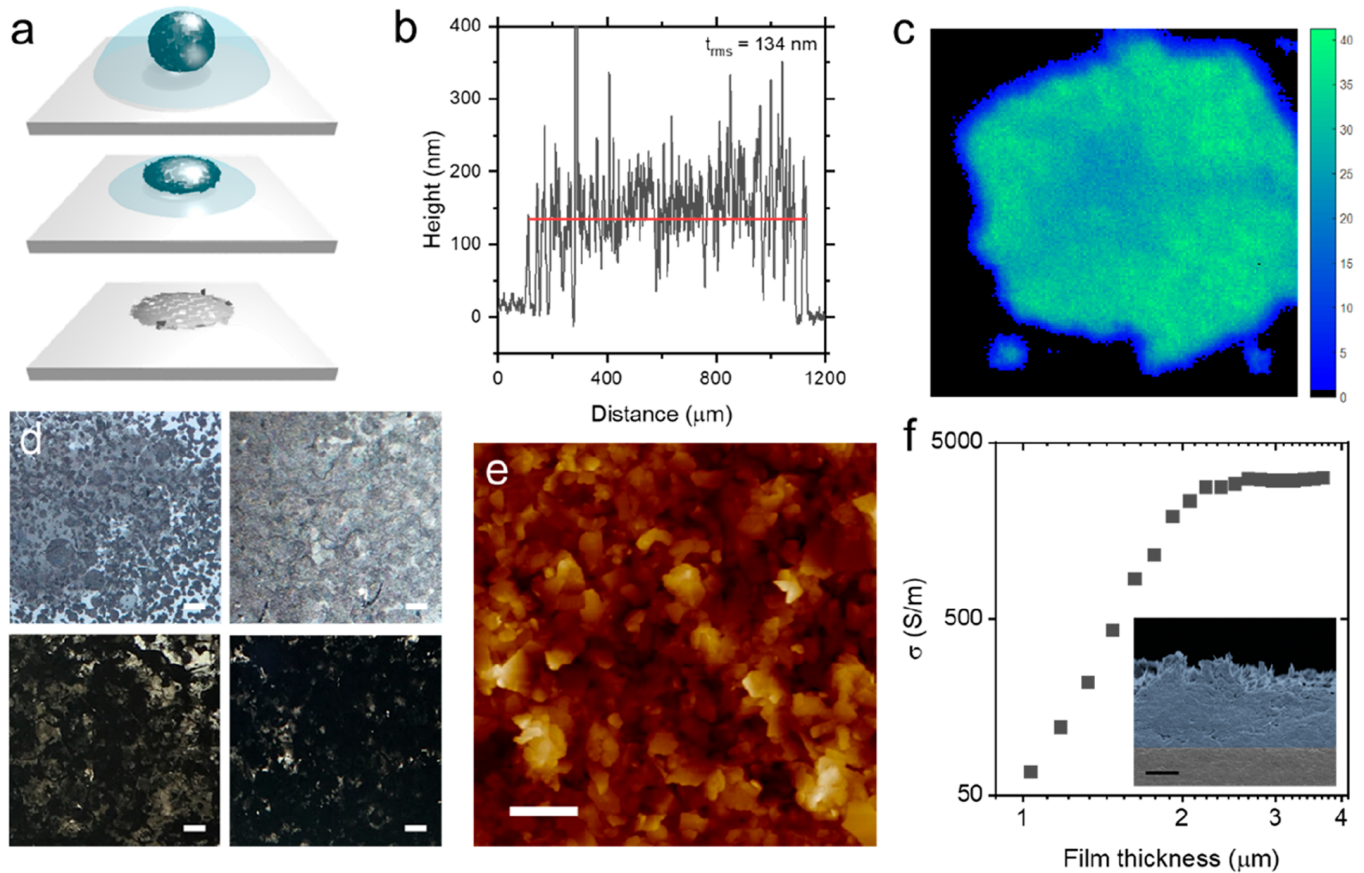
(a) Schematic illustration of emulsion droplet deposition, drying and collapse. (b) Stylus profilometry of deposited droplet showing film thickness of ∼130 nm, corresponding to ∼30 monolayers interfacial film thickness. (c) Raman map of G peak intensity illustrating the uniformity of deposited film; 30 × 30 μm image. (d) Low-magnification optical micrographs of deposited droplets on PET showing the sequential passes of emulsion deposition with percolation and formation of densely packed films; scale bars 500 μm. (e) Atomic force micrograph of nanosheet film confirming dense and uniform areal packing of the nanosheets deposited from a single emulsion droplet; scale bar 500 nm, height range 200 nm. (f) Electrical conductivity of the graphene film deposited from emulsion as a function of film thickness showing the scaling attributed to deposition uniformity, which reaches expected bulk-like value. Inset: Scanning electron micrograph of film cross section (false colored) showing the dense-packed nanosheet network; scale bar 1 μm.

The confinement of nanosheets at the liquid–liquid interface has the potential to enable controlled deposition of the interfacial films free from the drying dynamics which lead to the coffee ring effect when depositing from standard dispersions.^28^ In practice, droplets in water-in-cycloketone emulsions sediment onto the substrate but are stable in contact with hydrophobic polymeric substrates such as polyethylene terephthalate (PET), in contrast to glass where the droplets wet and spread or buoyant oil droplets which rise to the air interface and burst. This stability on hydrophobic substrates facilitates preferential evaporation of the solvent capping layer and confers a degree of spatial control to deposition even for manual dropwise deposition. As shown in Figure 2a, water droplets are stable on a substrate until spreading and evaporation of the capping layer of the solvent. The exposed graphene-coated water droplet then forms an unstable three-phase interface with the air (only stable for air-in-water), resulting in deformation, drying, and collapse of the droplet onto the substrate.

This controlled deposition of individual droplets also allows for the characterization of the interfacial films using techniques which are not possible in the emulsion structures. The deposited nanosheets are essentially collapsed bilayers of the disordered interfacial films which stabilize the emulsions, enabling verification of the thin-film structure such as by stylus profilometry. Figure 2b shows a representative height profile of a deposited droplet with ∼130 nm thickness. This can be interpreted as a nanosheet network with 50% porosity, that is, ∼60 nm equivalent nanosheet thickness, as a bilayer of the coating, suggesting <30 monolayers interfacial film thickness. This also has the potential to be tuned to control deposited film thickness as desired for a specific application. In addition, spectroscopic Raman mapping can be applied to deposited droplets to characterize material quality and uniformity. As shown in Figure 2c, the Raman G-peak intensity map (indicative of local graphene coverage) is extremely uniform across the droplet, confirming mitigation of the coffee ring effect which is prohibitive for deposition of standard depositions as microliter droplets.

The coffee-ring-free deposition of individual droplets suggests that it could be possible to assemble these deposited films into large-area nanosheet networks as solution-processed conductive films. To prepare such films, droplets were deposited manually in a dropwise manner, allowing them to dry before further deposition and repetition until the dense-packed networks were assembled, as shown in Figure 2d. These micrographs show areal increasing connectivity of the droplets as well as increasing film thickness as the deposited droplets are overcoated. The structure and thickness of these large-area films is elucidated by atomic force microscopy, as shown in Figure 2d, showing dense-packed nanosheets comparable to deposition by other techniques and allowing measurement of per-pass film thickness. The macroscopic conductivity of these films shown as a function of thickness for sequential deposition passes in Figure 2f. Interestingly, the conductivity only becomes measurable after a certain number of deposition passes where the thickness is around 1 μm, but areal percolation has only just been reached. The conductivity continues to increase from this onset value before saturating to a bulk-like constant value where the droplet-deposited network becomes densely packed, as shown in the inset scanning electron micrograph. Significantly, the maximum conductivity of ∼3000 S/m is comparable to other reported values for graphene networks but has been achieved with a simple manual deposition process with high material efficiency. As such, these emulsions could be developed as inks for printing of individual droplets with further development of their rheology to meet criteria for jetting (see Supporting Information Figure S6). While automated high-resolution deposition is required to eliminate the thickness dependence of the conductivity, the competitive conductivities illustrate the promise of nanosheet-stabilized emulsion deposition as a controlled low-mass low-volume technique for single-droplet and large-area film formation.

### Surface Energy: Determination and Design

**Figure 3:**
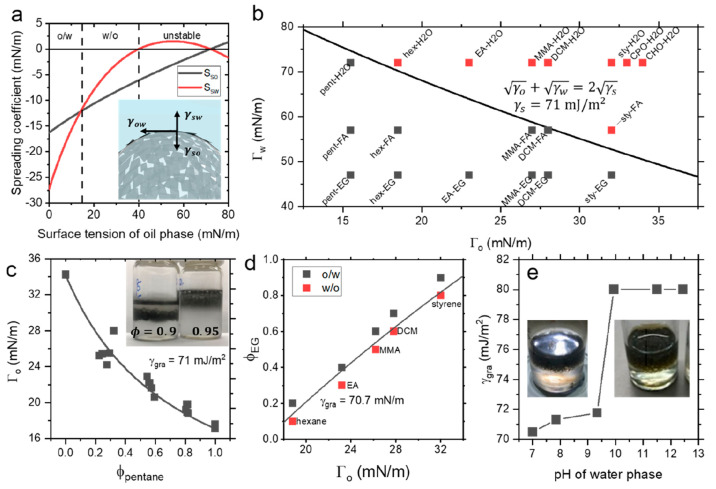
(a) Calculated spreading coefficients for emulsions of graphene and water (using γ = 70 and 101 mJ/m^2^, respectively) as a function of oil phase surface tension and the inset showing three-phase boundary at emulsion interface. (b) Surface tension phase diagram showing different compositions giving rise to w/o (black) and o/w (red) emulsions that is well described by eq 6 with a surface energy of ∼71 mJ/m^2^ for all pristine nanosheets studied here. (c) Surface tension of oil as a function of pentane volume fraction as an inversion experiment to determine surface energy, giving a value in good agreement with above measurements. (d) Volume fraction of ethylene glycol required for inversion as a function of oil phase surface tension for washed surfactant-exfoliated graphene, indicating that stabilization is still dictated by the nanosheets. (e) Nanosheet surface energy as a function of pH of the water phase, determined by pentane/CHO inversion. Inset: Photograph of buoyant cycloketone droplets in water continuous phase, inverted at elevated pH, shown for graphene (left) and MoS_2_ (right).

To realize the full range of applications envisaged, it will be necessary to form nanosheet-stabilized emulsions with liquids other than water and cycloketones. However, for the reasons illustrated in Figure 1a, it is quite challenging to use alternative solvents while retaining the high degree of exfoliation required for ultralow loading applications. In practice, this can be achieved using a solvent transfer step based on the liquid cascade centrifugation.^29^ Dispersions are prepared in cycloketones as normal and subjected to further centrifugation to sediment the majority of the nanosheets, the supernatant is discarded, and the sediment is redispersed into an alternative solvent of choice before immediate emulsification. This allows for production of well-exfoliated materials in solvents where this would not be possible by direct exfoliation, such that few-layer nanosheet-stabilized emulsions can be produced with relatively arbitrary oil and water phases. This approach allows emulsification of liquids with different surface tensions to modify the three-phase boundary shown in the inset of Figure 3a.

Emulsion stability and type, whether o/w or w/o, is conventionally determined by the spreading coefficient at this three-phase boundary, which is defined by the constituent interfacial energies as follows:
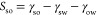
2
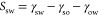
3where *S*_so_ and *S*_sw_ are the spreading coefficients for solid–oil and solid–water interfaces, respectively, and the subscripts of the interfacial energies denote the contributions as shown in Figure 3a. The criterion is typically that the spreading coefficients both have the same sign (positive or negative) for an emulsion to be stable. In addition, the phase with the more negative spreading preferentially wets the nanosheet stabilizer and therefore forms the continuous phase, while the other forms the droplet phase.^9^

While interfacial tensions between liquids can be measured, it would be preferable to understand the spreading coefficients as a continuous function of the individual and well-known surface tensions of the liquids. To facilitate this, well-established simple models for interfacial tension as a function of surface tension^19,30,31^ can be employed, such as the following geometric mean model:
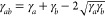
4For graphene and related materials, as solids, the surface entropy (and therefore surface tension Γ) is poorly defined, and therefore it is more correct to infer the surface energy γ from its interaction with liquids of known surface energy^24^ or by inverse gas chromatography.^32,33^ As such, liquid-exfoliated graphene is understood to have a surface energy close to 70 mJ/m^2^ based on good exfoliation and dispersion into solvents with surface tensions close to 40 mN/m. Using this indicative nanosheet surface energy and substituting eq 4 into eqs 2 and 3, it is possible to plot the spreading coefficients for two given phases as a function of the surface energy (or tension) of the third to assess stability and inversion criteria. Figure 3a shows the spreading coefficients for a system of graphene and water as a function of the surface tension of a third (oil) phase, which shows three distinct regions. Where the spreading coefficients have opposite signs (one positive, one negative), an emulsion would be unstable, and stable emulsions are only formed for γ_s_ > γ_o_ and, by extension, for γ_s_ < γ_w_, indicating that the stabilizer must have surface energy intermediate to the liquid phases. Where the spreading coefficients have the same sign (which only occurs when both are negative), whichever is more negative will form the continuous phase. This threshold can be elucidated by simplifying the spreading coefficients to give the intuitive criterion that phase inversion occurs when
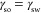
5As such, this inversion threshold can be further simplified, by substituting eq 4 into eq 5 (see Supporting Information) to give
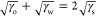
6where lower surface energies of the liquid phases give o/w and higher surface energies give w/o, and the threshold itself is determined by the surface energy of the solid stabilizer; in this case, the layered nanosheets. It should also be noted that the form of eq 6 is dictated by the geometric mean model used; however, we find that the harmonic mean model^31^ gives virtually identical numerical values and results in the same predictions for emulsion stability and type as eq 6.

This equation can be correlated with experimental observations of the emulsion type (w/o or o/w) to allow the determination of nanosheet surface energy and elucidate a phase diagram for the surface energy composition of an emulsion system. In practice, graphene and MoS_2_ are found to give the same emulsion type (w/o or o/w) for all combinations of immiscible liquids studied, and these are illustrated in Figure 3b. The inversion threshold in this phase diagram can be fitted to give the surface energy of the nanosheets as 71 mJ/m^2^. This is consistent with previous studies using other techniques suggesting that this emulsion inversion is indeed dictated by the nanosheet surface energy as described by eq 6. As such, this model of emulsion type in terms of constituent surface energy represents an approach both for the determination of unknown nanosheet surface energies and subsequently for the design and control of emulsion structures based on composition.

It should be noted that the nanosheet surface energy can only be determined to within the bounds dictated by these binary oil–water combinations which are susceptible to variation with any small miscibility between the phases. It is perhaps surprising that the surface energies of graphene and MoS_2_ cannot be differentiated by this binary oil–water approach; however, they are expected to be similar as a result of the interlayer binding energies characteristic of van der Waals bonding,^34^ and measuring any differences will be the subject of a follow-up study.

Here the objective is to demonstrate such an approach to identify practical strategies to control inversion to enable applications of emulsion networks as functional structures. From Figure 3b, it is clear that the need to control emulsion type could potentially compromise the range and practicality of compositions. Given that lower liquid phase surface energies promote an emulsion inversion from w/o to o/w, one option is to add a low surface energy organic to the existing cycloketone phase to reduce the oil phase surface energy and invert the emulsion. Figure 3c shows the results of this approach with the measured surface tension of the oil phase as a function of the pentane volume fraction. This in turn allows determination of the oil phase surface tension at the emulsion inversion threshold, which is observed to occur between pentane volume fractions of 0.9 and 0.95, as shown in the inset photographs. This gives the graphene surface energy as 71 mJ/m^2^, consistent with the binary combination approach, and highlights the high pentane volume fractions required to invert water-based emulsions stabilized by pristine nanosheets.

For most applications, it would be desirable to select the oil phase, for example, a solvent for deposition or monomer or polymer for composites, a phase change material, or other dispersed functional material, and form o/w emulsions to allow subsequent removal of the water phase. Figure 3b shows ethylene glycol (EG) as one of the only viable water phases which yields o/w emulsions for all oil phases studied, but its high boiling point can be problematic for subsequent removal. In addition, if the oil phase is dictated by the target application, rather than to allow pre-emulsification exfoliation, and the water phase is EG to realize o/w emulsions, both phases could be poor solvents for the retention of few-layer nanosheets and low-loading emulsions may not be possible. However, if the water phase could be a mixture of EG and water, the nanosheets could potentially be exfoliated in aqueous surfactant solution, washed, and transferred into this EG/water mixture to form o/w emulsions. Figure 3d shows the EG volume fraction in the EG/water mixture required to invert the emulsions formed from five different oil phases, using graphene nanosheets exfoliated in Triton X-100 surfactant solution. Importantly, these surfactant-exfoliated nanosheets can indeed stabilize emulsions, and their surface energies can be determined by fitting the EG volume fraction between the w/o and o/w emulsions. This fitting yields a surface energy of 70.7 mJ/m^2^, suggesting that the emulsion type is dictated by nanosheets and not influenced by any residual surfactant. This presents a promising strategy for the formation of nanosheet-stabilized emulsions with an arbitrary oil phase and controllable inversion.

Given the robustness of nanosheet surface energy to emulsion composition and surfactant addition, the influence of pH was investigated by analogy to the approach used for GO emulsions. At neutral pH, GO functional groups are deprotonated, increasing the polar contribution to the surface energy and promoting dispersion stability. Under acidic conditions, functional groups are reprotonated, reducing the polar contribution to the surface energy, destabilizing dispersions, and allowing emulsion formation. While edge functionalities of pristine nanosheets are poorly understood and challenging to investigate, their influence can be understood by such pH-dependent measurements. Polar functional groups would be expected to be protonated in organic solvents and surfactant solutions (reducing polarity and improving surface energy matching) but could be deprotonated under basic conditions to increase nanosheet surface energy and potentially enable emulsion inversion. Figure 3e shows the results of this approach where graphene surface energy is determined by the pentane mixing method as a function of the water phase pH (controlled by addition of KOH). It is important to note that, while ketones in basic conditions can undergo aldol condensation, the yield of such reactions would be small and unlikely to affect constituent surface energies and that this approach also works for nonketone oil phases. As shown by the inset photographs, emulsions of cycloketones and water at pH 13 clearly have buoyant (oil) droplets and have therefore undergone inversion to o/w as expected and intended. This is consistent with the observations from the pentane mixing experiments where the graphene surface energy increases with increasing pH, evidenced by the reducing pentane volume fraction required for inversion, before going through a step-change above pH 9, likely associated with deprotonation of edge functionalities, where all the cycloketone emulsions are o/w without pentane addition and the surface energy can be determined to be >80 mJ/m^2^. Interestingly, as shown by the inset photographs, this approach is transferable to MoS_2_ emulsions, suggesting some similarities in the pH response of their edge functionalities, likely thiol groups, which can also undergo deprotonation. This basic inversion can be performed with solvent- or surfactant-exfoliated nanosheets, presenting an all-water-based approach for emulsifying few-layer nanosheets and controlling the inversion of subsequent emulsions.

### Functional Emulsion Composites

To demonstrate the potential of this approach for the design and control for a range of compositions and applications, composites of graphene-stabilized silicone emulsions were investigated as a model system. Using silicone as the oil phase allows simple *in situ* curing to form solid composites with the potential for stretchable and flexible conductors and strain sensors. We have previously demonstrated graphene-stabilized emulsion composites using a viscous silicone which requires the addition of solvents to allow incorporation of graphene and emulsification; as a result, these composites require long interdiffusion and curing times and still require high graphene loadings as result of incomplete exfoliation in silicone-compatible solvents.^20^ Here, we utilize Ecoflex as a low-viscosity silicone, which can be directly emulsified, and surfactant-exfoliated nanosheets to preserve exfoliation and low-loading stabilization, by transferring into the EG water phase required for o/w emulsion formation.

**Figure 4:**
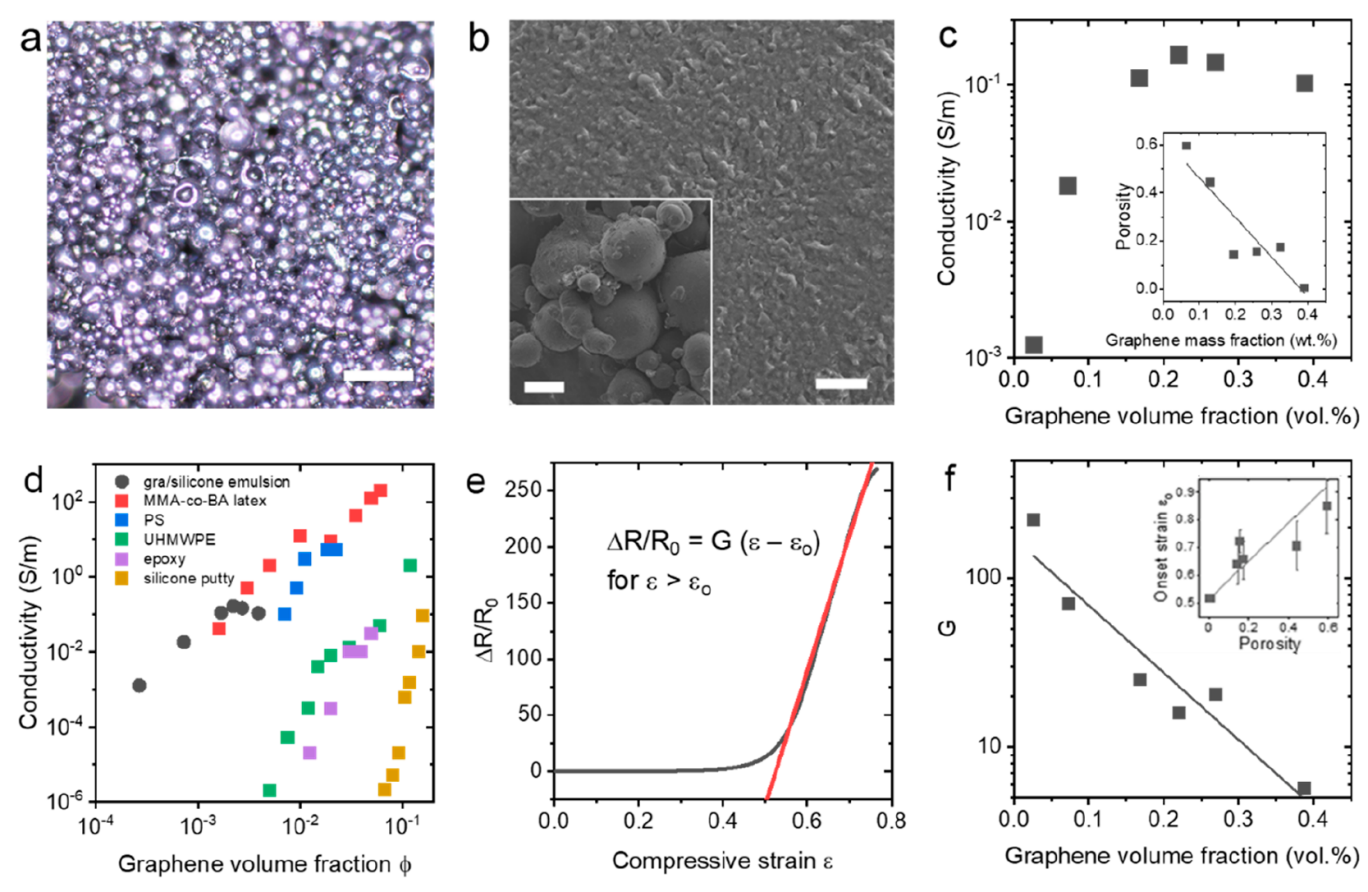
(a) Optical micrograph of emulsion-templated silicone composite shows graphene-coated surfaces and retained droplet structure; scale bar 200 μm. (b) Scanning electron micrographs of emulsion-droplet surfaces showing conductivity and graphene–polymer interface; scale bar 5 μm. Inset: Micrograph of emulsion composite showing droplet connectivity; scale bar 100 μm. (c) Electrical conductivity of composites as a function of loading level showing a sharp increase from lowest-loading high-porosity samples and saturation at higher loadings. Inset: Porosity of composites as a function of loading level determined by density measurements. (d) Conductivity-volume fraction comparison to composites from the literature based on pristine graphene in matrices of methyl methacrylate-butyl acrylate copolymer latex (MMA-*co*-BA latex),^39^ polystyrene (PS),^40^ ultrahigh molecular weight polyethylene (UHMWPE),^41^ epoxy,^42^ and silicone putty,^36^ highlighting the appreciable conductivity and ultralow loading level in the nanosheet-stabilized emulsion networks. (e) Representative relative resistance change as a function of compressive strain showing flat region associated with porosity, followed by a high-sensitivity linear region with potential for electromechanical sensing applications. (f) Compressive gauge factor extracted from the linear region showing high sensitivity decreasing exponentially with the loading level, as expected for nanocomposite sensors. Inset: Onset strain for the electromechanical response as a function of porosity showing linear correlation.

This allows for emulsions to be stabilized by few-layer nanosheets in arbitrary compositions to enable the formation of silicone emulsions at graphene loading levels as low as 0.065 wt %. Curing the silicone and removing the EG yield the emulsion-templated composites where partial interdiffusion of the polymer preserves the segregated network structures but forms cohesive yet porous composites, as shown in Figure 4a. Scanning electron microscopy (Figure 4b) shows that the droplet surfaces have appreciable conductivity and shows an apparently thin film of graphene at the polymer surface, as compared with previous work where droplet surfaces show thick graphitic networks.^20^ Significantly, despite their ultralow loading level, these composites exhibit an appreciable electrical conductivity, as shown in Figure 4c as a function of graphene volume fraction. The conductivity increases sharply from the lowest level, likely due to the reducing porosity, and saturates to around 0.1 S/m at higher loadings. Density measurements allow calculation of the porosity of the composites which is significant (>50%) at low loadings and decreases linearly at higher loadings, as shown in the inset of Figure 4c. Importantly, these conductivities and volume fractions, shown alongside the liquid emulsions and pristine graphene composites from the literature in Figure 4d, represent the lowest loading level ever reported for pristine graphene composites.

Furthermore, the soft porous nature of these conductive composites makes them well suited to compressive strain sensing.^35^ By compressing vertically and measuring the relative resistance change in the transverse direction, their electromechanical response can be elucidated. Figure 4e shows a representative relative resistance change *vs* compressive strain plot with a characteristic flat region associated with lack of electromechanical response as pores are eliminated without significant modification of the conductive network, followed by a high-sensitivity linear regime which allows for calibrated compressive strain sensing. From this, the compressive gauge factor is determined and is plotted against the loading level in Figure 4f, showing an exponential decrease from >200 with increasing loading level, typical of nanocomposite strain sensors.^36^ An onset strain for the linear response can be defined and is found to increase linearly with porosity (shown inset in Figure 4f). As such, these emulsion-templated silicone composites, especially at the lowest loadings, exhibit with a high electromechanical sensitivity which could be precompressed to facilitate low-applied-strain sensing with significant electromechanical response. Together, these results highlight the potential of emulsion-templated networks stabilized by few-layer nanosheets to enable ultralow loading conductive composites with tunable structure, electromechanical sensitivity, and the potential to extend to a range of other nanosheets, droplet phases, and applications.

## Conclusions

Nanosheet-stabilized emulsions represent a relatively unexplored approach for the formation of segregated networks where pristine few-layer 2D materials have the potential to confer emulsion stability and network conductivity at volume fractions as low as 10^–5^, approaching the minimum loading achievable with 2D nanosheets. In addition, liquid emulsions can be deposited as inks for functional thin films which preserve droplet structures and eliminate the coffee ring effect during drying. This controlled droplet deposition can be applied for single-droplet devices to allow thin-film formation from minute quantities of material or for sequential deposition of droplets to form large-area films with network conductivities competitive with other deposition techniques. The range of potential applications of nanosheet-stabilized can be broadened by investigation of emulsion composition and structure. By combining emulsion spreading coefficients and interfacial energy models, it is possible to understand emulsion stability and inversion from water-in-oil or oil-in-water in terms of the nanosheet and liquid surface energies. Importantly, this enables both the determination of nanosheet surface energies and the subsequent design of emulsions with controlled composition and structure. This approach can be applied to silicone as a model oil phase to prepare functional emulsion composites with record-low nanosheet loading levels and excellent electromechanical strain sensitivity. Together, these capabilities demonstrate the potential of nanosheet-stabilized emulsions for low-loading conductive networks, controlled droplet deposition, and surface energy determination and design for a broad range of functional segregated network applications.

## Experimental Section

### Exfoliation and Emulsification

Graphite powder was provided by Zenyatta Ventures Ltd. MoS_2_ and powder (98% purity) was purchased from Sigma-Aldrich. MoS_2_ was subjected to an initial sonication-centrifugation step to remove impurities and very small nanosheets. The bulk powder was added to 30 mL of cyclopentanone (CPO) at an initial concentration of 25 g/L and sonicated using a Sonic Vibra-cell VCX130 at 60% amplitude for 1 h under ice bath cooling. The dispersion was centrifuged (Thermo Scientific Sorvall Legend X1 with High Conic II rotor) at 5000*g* for 5 min, the supernatant containing the impurities and very small nanosheets was the discarded, and the sediment was redispersed into 30 mL of fresh CPO. Graphite powders were added to 30 mL of cyclohexanone at an initial concentration of 25 g/L. The subsequent sonication step used was the same for MoS_2_ and graphite; sonication using a Sonic Vibra-cell VCX130 at 60% amplitude for 3 h under ice bath cooling. MoS_2_ dispersions were centrifuged at 5000*g* for 5 min, and graphene dispersions were centrifuged at 5000*g* for 30 min. This typically yields dispersions of nanosheets with *N* < 10 for all materials, as confirmed with spectroscopic metrics by UV–vis extinction spectroscopy (Shimadzu UV3600Plus spectrometer). Extinction spectroscopy was also used in conjunction with previously measured extinction coefficients^26,37^ to determine concentration of these dispersions. Concentrations for these processing conditions are typically ∼0.1 g/L. These cycloketone dispersions can be emulsified with deionized water by transferring to silanized vials (Sigma-Aldrich) and adding water at ∼1:10 by volume followed by vigorously shaking by hand to homogenize. This gives nanosheet-stabilized water droplets which sediment through the cycloketone continuous phase. These droplets were collected and deposited on PET to perform statistical measurements of an average droplet diameter by optical microscopy (Olympus BX53-M optical microscope). To measure droplet size as a function of nanosheet volume fraction, the stock dispersions were diluted with cycloketone, and a fixed volume was emulsified with a fixed volume of water to control droplet size while maintaining a fixed volume of droplets. These samples were transferred into channels milled into PTFE with copper tape contacts to allow electrical measurements using a Keithley 2614B sourcemeter. *I*–*V* characteristics were obtained, and resistances normalized to the channel dimensions to calculate conductivity.

### Solvent Transfer and Emulsion Inversion

To prepare emulsions stabilized by well-exfoliated nanosheets in solvents which are conventionally considered poor for LPE, cycloketone dispersions were subjected to further centrifugation of 10,000*g* for 16 h to result in sedimentation of almost all the dispersed nanosheets. The cycloketone supernatant was discarded, and the sediment redispersed into a new oil phase such as pentane, hexane, ethyl acetate, methyl methacrylate, dichloromethane, or styrene. These oil phases span the range of surface energies of water-immiscible organic solvents and are immiscible with alternative high surface energy water phases; EG and formamide (except for ethyl acetate-formamide). As such, these combinations were used to identify emulsion orientation and stability. The solvent-transferred dispersions were emulsified with EG, formamide, and water at 1:1 by volume (to ensure sufficient oil and water phase to stabilize either orientation of the emulsion) and their orientation determined by identifying buoyancy and/or stability on glass or silanized vials or at the air interface. These orientations were used to verify the surface energy model presented and found to be identical for graphene and MoS_2_ emulsions whether exfoliated or bulk material was used. To perform the inversion experiment, a CHO dispersion was diluted to varying volume fractions of pentane, and the mixed solvent dispersion emulsified with water and orientation determined. Samples between which the emulsion orientation inverted were used to calculate a range for the surface energy of the nanosheet films.

### Emulsification by Surfactant-Exfoliated Nanosheets and Basic Inversion

For the emulsification of surfactant-exfoliated nanosheets, dispersions were prepared using the exfoliation parameters described above on dispersions of graphene or MoS_2_ in 0.25 g/L aqueous Triton X-100 solution, which yields a dispersion with a minimal amount of surfactant, likely bound to the sheets rather than free in dispersion. Surfactant concentration of 0.1 g/L was found to result in significantly reduced concentration, while dispersions produced by exfoliation at higher surfactant concentrations required washing by vacuum filtration and redispersion to allow stable emulsification. For the emulsion inversion by basic deprotonation, cycloketone dispersions were prepared, emulsified with pH 13 KOH solution, and diluted to yield water phases with controlled pH, resulting in formation of buoyant oil droplets in a continuous phase of the basic solution above pH 9. Surfactant exfoliation and basic inversion can also be achieved by blending aqueous surfactant dispersions of nanosheets with KOH solution followed by emulsification with an arbitrary oil phase.

### Emulsion Inks

Water-in-cycloketone emulsions of graphene and MoS_2_ were prepared as described above. Samples were deposited by onto the PET substrate heated to 80 °C by manual drop casting of 0.1 mL (per pass) of densely packed emulsion over an area of 1 cm^2^. The sheet resistance was measured using a Keithley 2614B sourcemeter after every deposition pass. Once dry, another 0.1 mL was deposited, and this was repeated until optical microscopy showed the films to have nearly complete area coverage, around 5 passes. At this stage, AFM was performed using a Bruker Dimension Icon with ScanAsyst-Air probes to measure topography and determine the approximate thickness per pass. For Raman mapping of deposited droplets, samples were deposited onto silicon wafers, and their Raman spectra were mapped using a Renishaw inVia Raman microscope with 660 nm excitation using a 50× objective. The deposition process was repeated until the sheet resistance began to decrease with the reciprocal of pass number, indicating that the thickness-independent bulk-like conductivity regime had been reached. Single deposited droplet thickness measurements were performed with a Bruker Dektak stylus profilometer.

### Silicone Composites

Few-layer graphene dispersions in aqueous Triton X-100 solution were prepared as described in our previous work.^38^ The few-layer graphene supernatant was centrifuged at 5000*g* for 16 h to sediment the majority of the dispersed graphene. The supernatant was discarded along with any unbound surfactant, and ethylene glycol was added to the sediment and redispersed *via* tip sonication at high concentration ∼3 g/L, forming the stock dispersion. This highly concentrated graphene-EG dispersion was increasingly diluted with additional EG. Ecoflex 00-30 was prepared from a two-part mix by stirring for about 1 min before being added to each graphene-EG dispersion. Each sample was immediately shear mixed at 10,000 rpm (Silverson L5M-A) for 2 min and cast into a glass Petri dish. The samples are placed in a preheated oven at 70 °C overnight, which is sufficient to cure the silicone spheres and remove the continuous EG phase. Raman spectroscopy was performed using a 532 nm laser; through an 1800 mm^–1^ grating under a 20× objective at 10% power (3.5 mW) with an exposure time of 0.5 s and 1 μm step size. For the electromechanical measurements, (∼5–7 mm × 20 mm), samples are cut and placed on glass for insulation and mechanical support. Silver contacts are painted on the ends of the sample, connected in series to a Keithley 2614B sourcemeter for electrical characterization. A 1 × 1 cm^2^ glass slide was attached to the base of a compression probe (Texture Analyzer, Stable Microsystems) such that the unpainted area of the sample was subject to compression and to prevent shorting through the metal probe.

## Supporting Information Available

Video S1: Emulsion droplets showing controlled coffee-ring-free deposition during drying (MOV)
Video S2: Emulsion droplets deposited onto narrow-gap electrodes to illustrate potential for single-droplet devices (MOV)
Statistical Raman spectroscopy of nanosheets; emulsion droplet micrographs, size distributions, stability, and conductivity data; emulsion droplet size model; characterization of deposited emulsion droplets; derivation of surface energy model for emulsions; rheological measurements; droplet conductivity model; Raman and electromechanical characterization of silicone emulsion composites (PDF)

## Supplementary Material

nn1c06519_si_001.mov

nn1c06519_si_002.mov

## Notes

A version of this manuscript was previously submitted as a preprint: Ogilvie, S. P.; Large, M. J.; Cass, A. J.; Amorim Graf, A.; Sehnal, A. C.; O’Mara, M. A.; Lynch, P. J.; Salvage, J. P.; Alfonso, M.; Poulin, P.; King, A. A. K.; Dalton, A. B. Nanosheet-stabilized emulsions: ultralow loading segregated networks and surface energy determination of pristine few-layer 2D materials. *arXiv (Applied Physics)*, May 13, 2020, 2005.06330, ver 1. https://arxiv.org/abs/2005.06330 (accessed 2022-01-25).
