# Supplementary material for: Nanosheet-Stabilized Emulsions: Near-Minimum Loading and Surface Energy Design of Conductive Networks
Source: ACS Nano. 2022 Feb 2;16(2):1963–73. doi: 10.1021/acsnano.1c06519 (PMC9007533; doi:10.1021/acsnano.1c06519)
Supplement: Supplementary file 3 — nn1c06519_si_003.pdf [file nn1c06519_si_003.pdf]

Supporting Information for

Nanosheet-Stabilized Emulsions: Near-Minimum Loading and Surface Energy Design of Conductive Networks

Sean P. Ogilvie[a]*, Matthew J. Large[a], Marcus A. O'Mara[a], Anne C. Sehnal[a], Aline Amorim Graf[a], Peter J. Lynch[a], Adam J. Cass[a], Jonathan P. Salvage[b], Marco Alfonso[c], Philippe Poulin[c], Alice A. K. King[a] and Alan B. Dalton[a]*

[a] University of Sussex, Brighton, BN1 9RH, United Kingdom

[b] University of Brighton, Brighton, BN2 4GJ, United Kingdom

[c] Centre de Recherche Paul Pascal - CNRS, University of Bordeaux, 33600 Pessac, France

* s.ogilvie@sussex.ac.uk; a.b.dalton@sussex.ac.uk

Statistical Raman characterisation

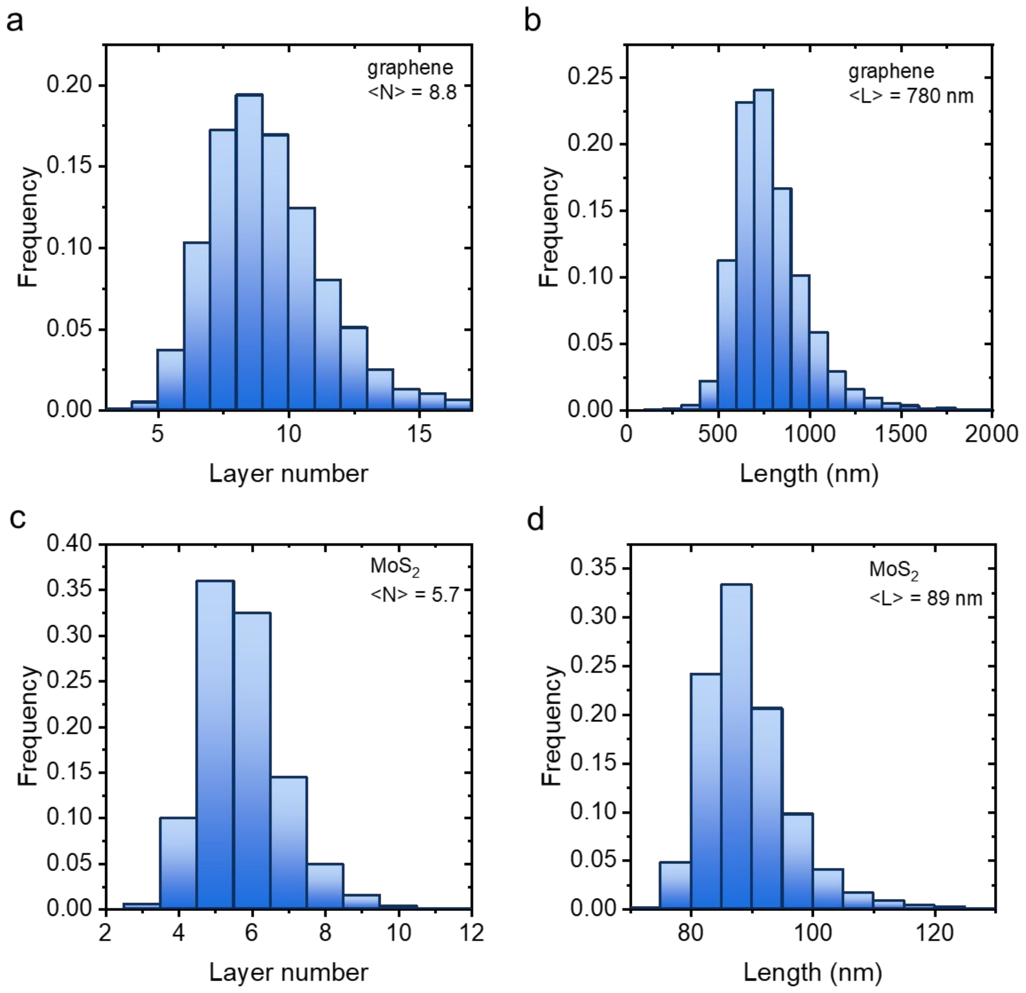

Figure S1: Histograms of graphene layer number (a) and length (b) and MoS_2 layer number (c) and length (d) based on established Raman spectroscopic metrics and statistical mapping[1,2], with calculated pixel-weighted averages also given.

Droplet micrographs and conductivity

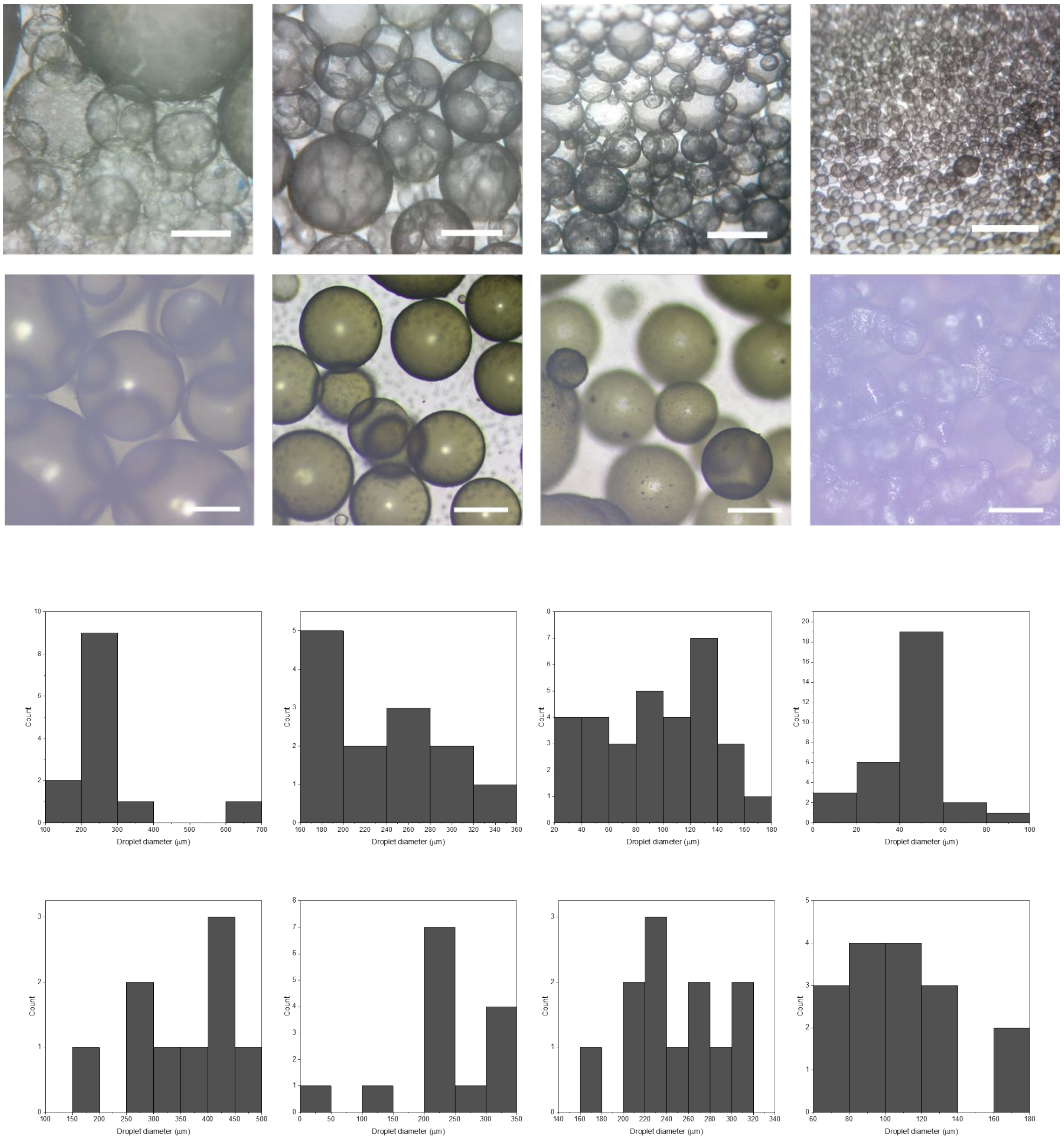

Figure S2: Optical micrographs for emulsion droplet size characterization, top row graphene, second row MoS_2, left to right, increasing nanosheet volume fraction, scale bars 200 μm. Below are corresponding droplet size distributions showing broad distributions with some evidence of multi-modality at lower loadings but narrowing of distributions at higher loadings.

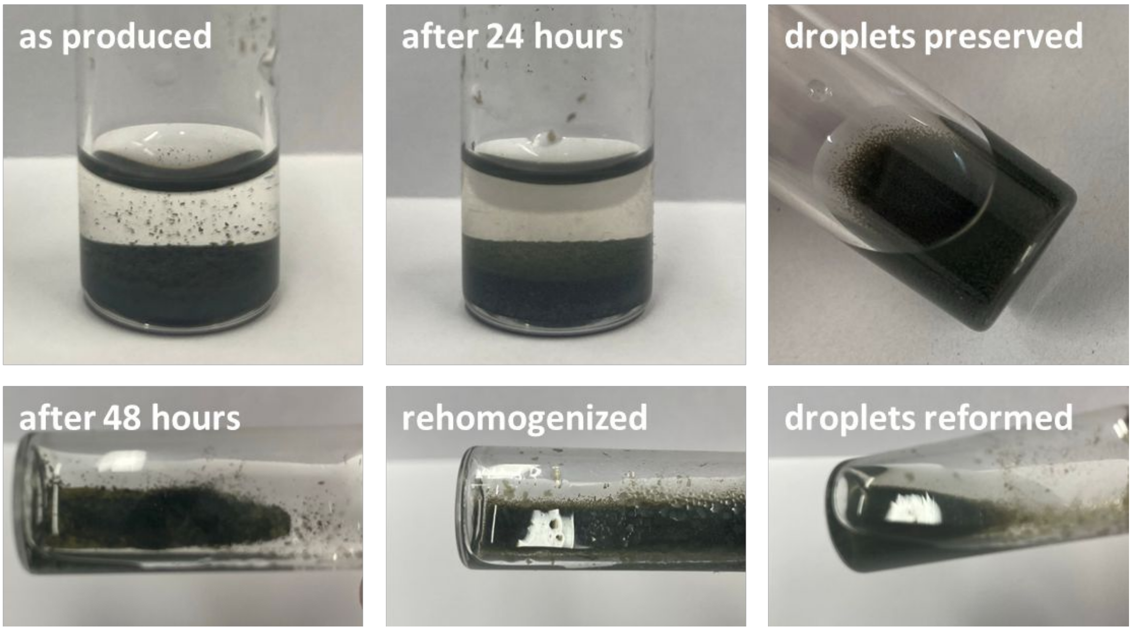

Figure S3: Photographs illustrating emulsion stability showing stable sedimented droplets immediately after production, after 24 hours with clear discrete droplets. After 48 hours, there is some coalescence, however the emulsion can be rehomogenized to reform stable droplets without aggregation or loss of surface area.

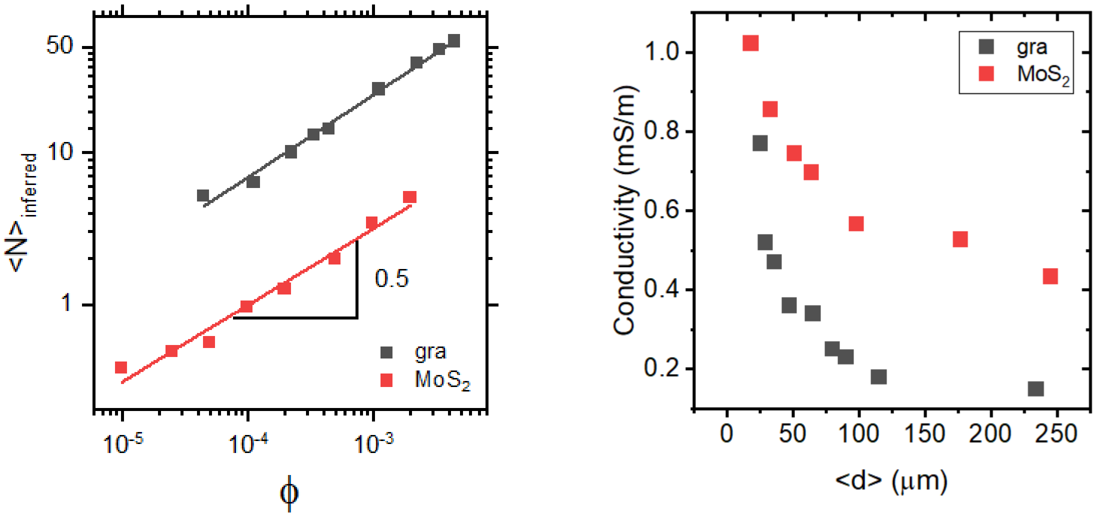

Figure S4: (left) Estimated layer number from geometric model using droplet size and volume fraction, indicating few-layer nature of interfacial films. (right) Emulsion droplet network conductivity as a function of emulsion droplet diameter showing smaller droplets sizes (at lower

loading levels) for MoS_2 compared with graphene, likely contributing to increased conductivity through improve network packing and increased parallelization.

Droplet size model

Droplet size can be related to nanosheet volume fraction (relative to the droplet phase) by equating the surface area of the droplets to that of the nanosheets. The surface area A of a droplet can be related to its diameter $\langle d \rangle$ and the mass m, specific surface area SSA and thickness as a number of monolayers of the nanosheets $\langle N \rangle$ as

$$A = \pi \langle d \rangle^2 = \frac{m\,SSA}{\langle N \rangle} \tag{1}$$

The mass of stabilising nanosheets can be related to their volume fraction by

$$m = \phi \rho_{2D} \pi \langle d \rangle^3 / 6 \tag{2}$$

By combining the above

$$\pi \langle d \rangle^2 = \frac{\phi \rho_{2D} \pi \langle d \rangle^3 \, SSA}{6 \langle N \rangle} \tag{3}$$

Noting that for layered materials the density and specific surface area can be related to the interlayer spacing as $c_{2D} = 1/\rho_{2D} SSA$, the above can be simplified to give a simple expression relating droplet diameter to nanosheet volume fraction

$$\langle d \rangle = \frac{6 c_{2D} \langle N \rangle}{\phi} \tag{4}$$

Controlled droplet deposition

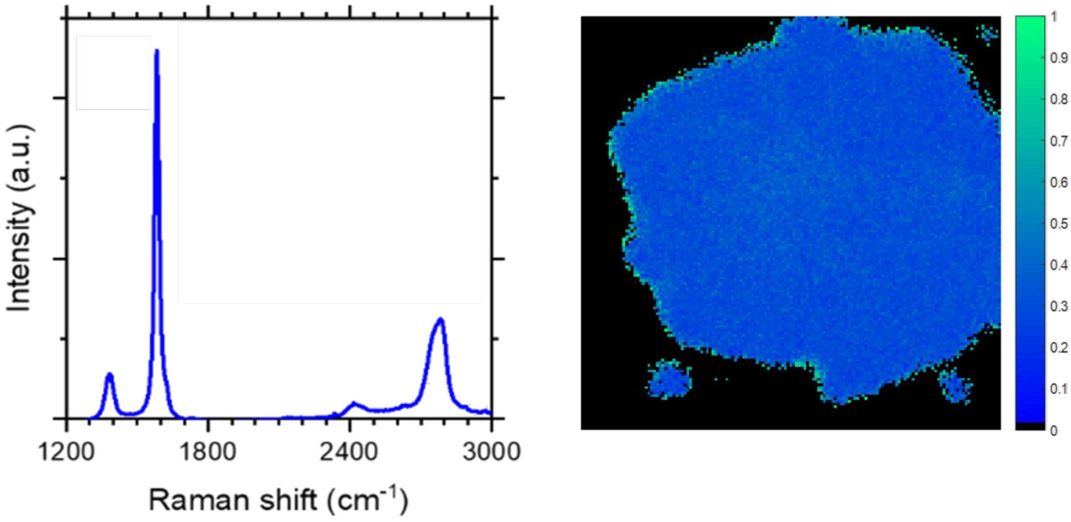

Figure S5: (left) Raman spectrum of deposited emulsion droplet and (right) Raman mapping of 2D/G peak ratio indicating few-layer graphene character and uniformity across droplet.

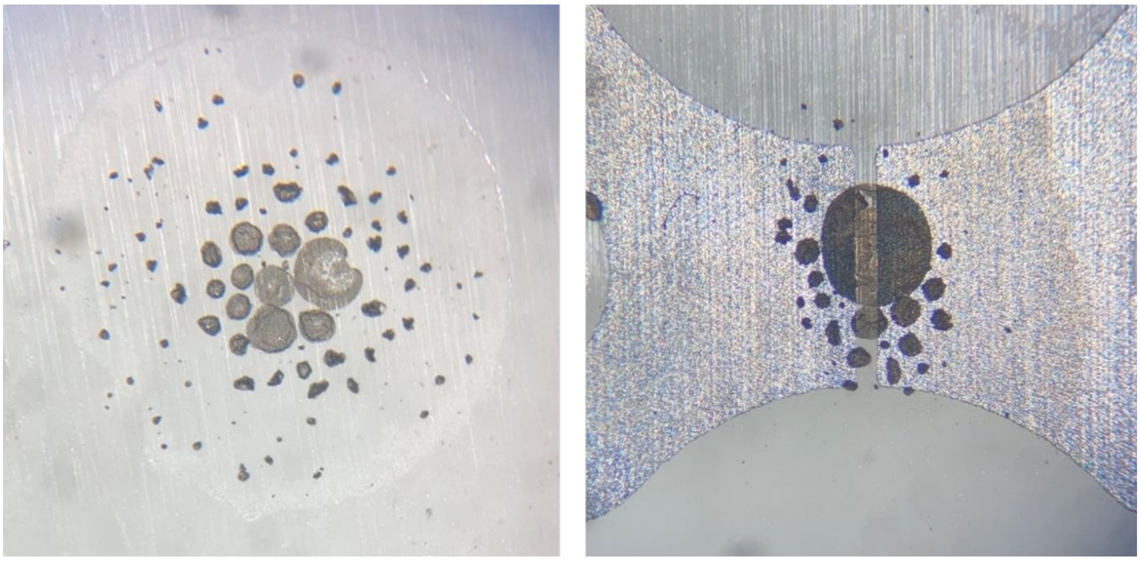

Figure S6: Optical micrographs taken from supporting video files showing (left) deposited droplets and (right) assembly across narrow electrode gap (~100 µm), illustrating potential for single-droplet devices.

Surface energy model

The stability and orientation of solid-stabilized emulsions can be related to the spreading coefficients and constituent interfacial energies. The spreading coefficients for the solid-oil and solid-water are given by

$$S_{so} = \gamma_{so} - \gamma_{sw} - \gamma_{ow} \quad (5)$$

$$S_{sw} = \gamma_{sw} - \gamma_{so} - \gamma_{ow} \quad (6)$$

Where the γ_{so}, γ_{sw} and γ_{ow} are the interfacial energies at the solid-oil, solid-water and oil-water interfaces. The above definitions can be combined to give

$$S_{so} + S_{sw} = -2\gamma_{wo} \quad (7)$$

Since interfacial tensions/energies are positive, spreading coefficients can only have the same sign (and thereby form a stable emulsion) if that sign is negative. If both spreading coefficients are negative, the stability criteria can be expressed as

$$\gamma_{so} - \gamma_{sw} < \gamma_{wo} \quad (8)$$

$$\gamma_{sw} - \gamma_{so} < \gamma_{wo} \quad (9)$$

Since $S_{so} - S_{sw} = -(S_{sw} - S_{so})$, one of the above equations will always be satisfied and the criterion reduces to

$$|\gamma_{so} - \gamma_{sw}| < \gamma_{ow} \quad (10)$$

Based on the geometric and harmonic mean models, it can be intuitively argued that it is most easily satisfied by $\gamma_o \ll \gamma_w$ (giving large γ_{ow}) and $\gamma_s \approx \gamma_o$and $\gamma_s \approx \gamma_w$ (giving $\gamma_{so} \approx \gamma_{sw}$) and the difference is small), which requires that $\gamma_o < \gamma_s < \gamma_w$, as illustrated in Fig. 4a, although this is more challenging to demonstrate rigourously.

However, in order to explicitly state this condition, interfacial energy models are required. The orientation of an emulsion (o/w or w/o) is also determined by the spreading coefficients, *i.e.*

whichever is more negative forms the droplet phase; o/w for $S_{so} < S_{sw}$ and w/o for $S_{so} > S_{sw}$. As such, the point at which they are equal can be considered the inversion threshold for an emulsion. This can be simplified (by definition and without any empirical models) as

$$\gamma_{so} = \gamma_{sw} \tag{11}$$

Subsequently, simple models for interfacial energies can be substituted such as[3]

$$\gamma_{ab} = \gamma_a + \gamma_b - 2\sqrt{\gamma_a \gamma_b} \tag{12}$$

$$\gamma_{ab} = \gamma_a + \gamma_b - 4\frac{\gamma_a \gamma_b}{\gamma_a + \gamma_b} \tag{13}$$

Incorporating the geometric mean model (Equation 12) into Equation 11 gives an expression which describes the inversion threshold of emulsions as a function of the constituent surface energies

$$\gamma_s + \gamma_o - 2\sqrt{\gamma_s \gamma_o} = \gamma_s + \gamma_w - 2\sqrt{\gamma_s \gamma_w} \tag{14}$$

$$\gamma_o - 2\sqrt{\gamma_s \gamma_w} = \gamma_w - 2\sqrt{\gamma_s \gamma_w} \tag{15}$$

Substituting $\gamma_o = x^2$ and $\gamma_w = y^2$

$$x^2 - 2\sqrt{\gamma_s}x = y^2 - 2\sqrt{\gamma_s}y \tag{16}$$

$$x^2 - y^2 = 2\sqrt{\gamma_s}x - 2\sqrt{\gamma_s}y \tag{17}$$

$$(x - y)(x + y) = 2\sqrt{\gamma_s}(x - y) \tag{18}$$

Cancelling $(x - y)$ gives

$$x + y = 2\sqrt{\gamma_s} \tag{19}$$

Finally, re-expressing in terms of surface energies yields

$$\sqrt{\gamma_o} + \sqrt{\gamma_w} = 2\sqrt{\gamma_s} \tag{20}$$

Non-Newtonian rheology

Proof-of-concept rheological measurements were performed to demonstrate the non-Newtonian behaviour of these nanosheet-stabilized emulsions. Shear-rate dependent viscosity is shown for a representative graphene-stabilized water-in-CHO emulsion. By performing measurements at different gaps between plates, we find that larger gaps where the droplets are not in contact with the top plate yield low viscosity and Newtonian behaviour. For the smaller gaps where the droplets are in contact with the top plate, we observe reproducible non-Newtonian rheology with characteristic power law behavior scaling $\eta \approx 0.1\,\dot{\gamma}^{-0.5}$, suggesting that the emulsion structure and therefore the nanosheet stabilizer is critical to realizing tunable viscosity for shear-based deposition techniques. In addition, while there is some coalescence into larger droplets during the rheological measurements, the emulsion structure is largely preserved.

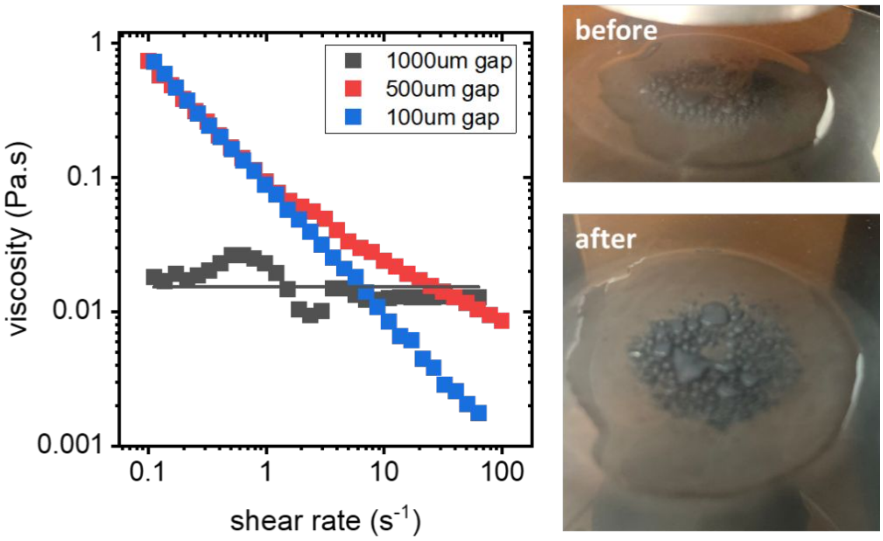

Figure S7: (lef) viscosity as a function of shear rate during parallel plate rheology of representative graphene-stabilized water-in-CHO emulsion and (right) photograph of emulsion before and after measurement, show some coalescence under shear but preservation of the droplets.

In order to be suitable for inkjet deposition, these emulsions also require a viscosity (~0.01 Pa.s) greater than that of common solvents at the shear rates applied during jetting (~10^6 s^{-1}). While these water-in-cycloketone emulsions reach the viscosity required for inkjet printing at 100 s^{-1}, 10^4 times

lower shear rate than during jetting, it it possible that viscosity will rapidly saturate at higher shear rates as shown previously for clay-stabilized water-in-oil emulsions[4]. Alternatively, it may be possible to use dilute emulsions (with lower ratio of droplet to continuous phase) which are known to exhibit Newtonian behaviour with viscosity independent of shear rate[5] to ensure the desired viscosity during jetting. However, this does mean reducing the concentration of the emulsion ink and potentially using a high viscosity (likely high boiling point) continuous phase, the selection of which must also satisfy other criteria for surface energy, nanosheet dispersability, *etc*. A more practical alternative might be to manipulate the shear rate-dependent viscosity by controlling emulsion droplet size. It is well known that smaller droplets in a concentrated emulsion give rise to increased viscosity[5,6] which presents a route to ensure sufficient viscosity during inkjet.

Droplet conductivity model

It is possible to develop a simple model for the resistor network of the system and its variation with droplet size which is in turn a function of volume fraction. A network of emulsion droplets can be approximated by resistors between droplets (R_j) connected by two resistors in parallel corresponding to droplet surface (R_s) and through-droplet (R_d) conductivity as shown in Figure S4.

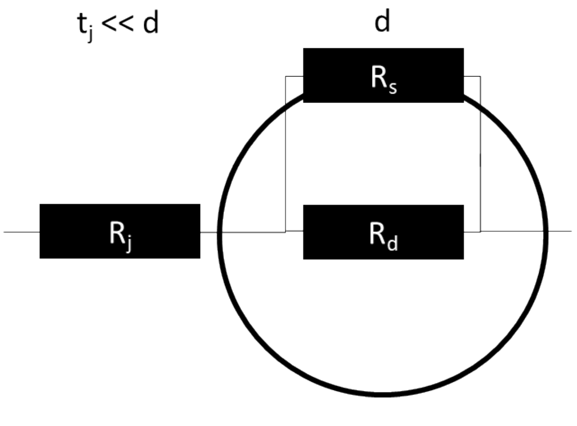

Figure S8: Unit cell of droplet network in simple conductivity model.

A two-dimensional projection of this "unit cell" as a square of side length d, with junction and surface thicknesses t_j and t_s, allows calculations of the total resistance and normalisation of the unit cell geometry.

$$R_t = R_j + \frac{1}{(\frac{1}{R_s} + \frac{1}{R_d})} \tag{21}$$

$$R_t = R_j + \frac{R_s R_d}{(R_d + R_s)} \tag{22}$$

This total unit cell resistance can be related to the conductivity and dimensions of the constituent phases and subsequently equated to the conductivity and dimensions of the unit cell itself as

$$R_t = \frac{t_j}{\sigma_j d^2} + \frac{\frac{1}{\sigma_s t_s} \cdot \frac{1}{\sigma_d d}}{(\frac{1}{\sigma_d d} + \frac{1}{\sigma_s t_s})} = \frac{t_j}{\sigma_j d^2} + \frac{1}{\sigma_s t_s + \sigma_d d} \equiv \frac{1}{\sigma d} \tag{23}$$

$$\sigma = \left[d(\frac{t_j}{\sigma_j d^2} + \frac{1}{\sigma_s t_s + \sigma_d d}) \right]^{-1} \tag{24}$$

$$\sigma = \frac{1}{d}(\frac{t_j}{\sigma_j d^2} + \frac{1}{\sigma_s t_s + \sigma_d d})^{-1} \tag{25}$$

Depending on whether the conduction is dominated by the droplets and surfaces or the junctions, this model will be dominated by the former or latter terms respectively. Where the droplets are much more conductive than the junctions, such as for water droplets stabilized by thick conductive nanosheet films in a very insulating oil phase, this leads to a decreasing conductivity with increasing loading, as observed in our previous work[7]

$$\frac{1}{\sigma_s t_s + \sigma_d d} \rightarrow 0 \quad (26)$$

$$\sigma = \frac{1}{d}\frac{\sigma_j d^2}{t_j} = \frac{\sigma_j d}{t_j} \quad (27)$$

By contrast, for coalesced emulsion polymer composites, where any inter-droplet junction resistances are reduced, this model simplifies to give a linear increase in conductivity with loading level

$$\frac{t_j}{\sigma_j d^2} \rightarrow 0 \quad (28)$$

$$\sigma = \frac{\sigma_s t_s + \sigma_d d}{d} \quad (29)$$

$$\sigma = \frac{\sigma_s t_s}{d} + \sigma_d \quad (30)$$

$$\sigma = \frac{\sigma_s}{6}\phi + \sigma_d \quad (31)$$

In practice, the all-liquid emulsion networks studied in this manuscript exhibit some intermediate behaviour which can be fitted to the original model but is also functionally equivalent to a power law in the range studied, as shown in Fig. 2c.

Silicone composites

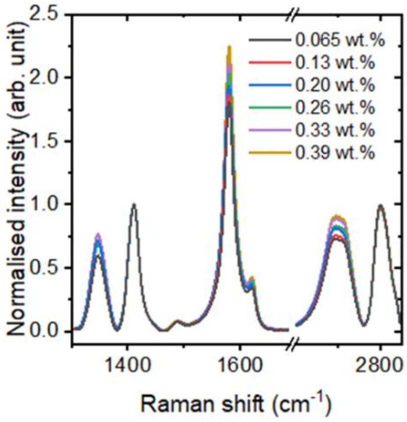

Figure S9: Raman spectra of graphene-stabilized silicone emulsion composites, normalized to silicone peak position showing increasing G peak intensity associated with increasing loading level.

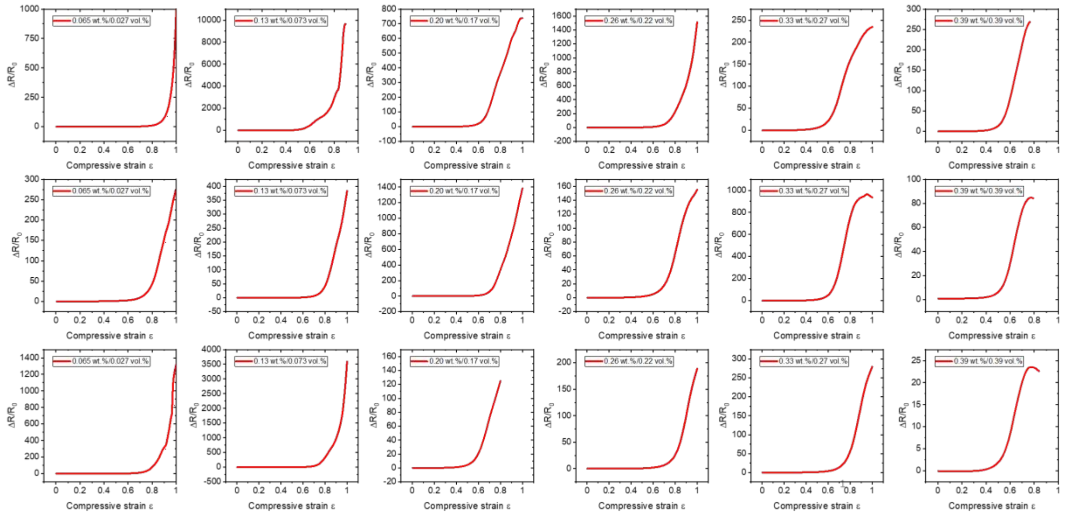

Figure S10: Relative resistance change as a function of compressive strain for three different samples of each loading level of emulsion-templated graphene-silicone composites, showing well-defined response with flat insensitive region associated with porosity, followed by highly-sensitive linear regime which facilitates electromechanical sensing.

References

(1) Backes, C.; Paton, K.; Hanlon, D.; Yuan, S.; Katsnelson, M.; Houston, J.; Smith, R.; McCloskey, D.; Donegan, J.; Coleman, J. N. Spectroscopic Metrics Allow in Situ Measurement of Mean Size and Thickness of Liquid-Exfoliated Graphene Nanosheets. *Nanoscale* **2016**, *8*, 4311–4323.

(2) Amorim Graf, A.; Ogilvie, S. P.; Wood, H. J.; Brown, C. J.; Tripathi, M.; King, A. A. K.; Dalton, A. B.; Large, M. J. Raman Metrics for Molybdenum Disulfide and Graphene Enable Statistical Mapping of Nanosheet Populations. *Chem. Mater.* **2020**, *32* (14), 6213–6221. https://doi.org/10.1021/acs.chemmater.0c02109.

(3) Fowkes, F. M. Determination of Interfacial Tensions, Contact Angles, and Dispersion Forces in Surfaces by Assuming Additivity of Intermolecular Interactions in Surfaces. *J. Phys. Chem.* **1962**, *66* (2), 382–382. https://doi.org/10.1021/j100808a524.

(4) Binks, B. P.; Clint, J. H.; Whitby, C. P. Rheological Behavior of Water-in-Oil Emulsions Stabilized by Hydrophobic Bentonite Particles. *Langmuir* **2005**, *21* (12), 5307–5316. https://doi.org/10.1021/la050255w.

(5) Derkach, S. R. Rheology of Emulsions. *Adv. Colloid Interface Sci.* **2009**, *151* (1), 1–23. https://doi.org/10.1016/j.cis.2009.07.001.

(6) Pal, R. Effect of Droplet Size on the Rheology of Emulsions. *AIChE J.* **1996**, *42* (11), 3181–3190. https://doi.org/10.1002/aic.690421119.

(7) Large, M. J.; Ogilvie, S. P.; Meloni, M.; Graf, A. A.; Fratta, G.; Salvage, J.; King, A. A. K.; Dalton, A. B. Functional Liquid Structures by Emulsification of Graphene and Other Two-Dimensional Nanomaterials. *Nanoscale* **2018**. https://doi.org/10.1039/C7NR05568D.
